# Sepsis: Precision-Based Medicine for Pregnancy and the Puerperium

**DOI:** 10.3390/ijms20215388

**Published:** 2019-10-29

**Authors:** Orene Greer, Nishel Mohan Shah, Shiranee Sriskandan, Mark R. Johnson

**Affiliations:** 1 Imperial College London, Academic Department of Obstetrics & Gynaecology, Level 3, Chelsea & Westminster Hospital, 369 Fulham Road, London SW10 9NH, UK; o.greer@imperial.ac.uk (O.G.); nishel.shah@imperial.ac.uk (N.M.S.); 2 Chelsea & Westminster Hospital, 369 Fulham Road, London SW10 9NH, UK; 3 Imperial College London, NIHR Health Protection Research Unit in Healthcare Associated Infections and Antimicrobial Resistance, Department of Infectious Disease, Imperial College London, Hammersmith Campus, Du Cane Road, London W12 0NN, UK; s.sriskandan@imperial.ac.uk

**Keywords:** sepsis, pregnancy, immunology, cardiovascular, transcriptomics

## Abstract

Sepsis contributes significantly to global morbidity and mortality, particularly in vulnerable populations. Pregnant and recently pregnant women are particularly prone to rapid progression to sepsis and septic shock, with 11% of maternal deaths worldwide being attributed to sepsis. The impact on the neonate is considerable, with 1 million neonatal deaths annually attributed to maternal infection or sepsis. Pregnancy specific physiological and immunological adaptations are likely to contribute to a greater impact of infection, but current approaches to the management of sepsis are based on those developed for the non-pregnant population. Pregnancy-specific strategies are required to optimise recognition and management of these patients. We review current knowledge of the physiology and immunology of pregnancy and propose areas of research, which may advance the development of pregnancy-specific diagnostic and therapeutic approaches to optimise the care of pregnant women and their babies.

## 1. Introduction

Sepsis in pregnancy is an important cause of maternal death [1]. In the United States (US) the incidence of severe maternal sepsis is increasing, and patients with complex medical problems are at the greatest risk [2,3]. Current management strategies include early detection, prompt initiation of resuscitation and anti-pathogen treatment. However, in pregnancy, sepsis is a complex pathogenic process compounded by immune-modulatory adaptations unique to pregnancy. The field requires innovation in early diagnosis, point of care tests and targeted therapy.

In this review, we aim to highlight the impact of sepsis on maternal morbidity and mortality worldwide; to summarise the physiological and immunological features unique to pregnancy that may impact on the development of sepsis and to explore research methods, which may help identify predictive and prognostic biomarkers for this patient population. We will answer the question of how physiological and immunological adaptations in pregnancy alter the maternal response to sepsis, and how this may influence the development of prognostic and therapeutic targets for this patient group.

## 2. Maternal Sepsis

### 2.1. The Global Picture

The impact of sepsis is of global importance. Accurate morbidity and mortality statistics are currently elusive, but it is estimated that 30 million patients worldwide are affected each year and that 6 million of these die [4,5]. Sepsis is defined as a dysregulated host response to infection leading to organ dysfunction. However, our knowledge of the underlying pathophysiology is incomplete; this is a significant contributing factor in our inability to deal effectively with this health epidemic.

Low and middle-income countries are disproportionately affected by the condition and particularly vulnerable populations include those with co-morbidities, the elderly, children as well as pregnant and puerperal women. 1 in 10 maternal deaths worldwide are attributed to sepsis [5,6] and 1 million neonatal deaths are secondary to maternal infection, including maternal sepsis [5,7].

Definitive evidence of increased susceptibility to sepsis in pregnancy is limited and a retrospective national study of age-matched pregnant and non-pregnant women in the US demonstrated a higher sepsis-related case fatality in the non-pregnant group [8]. This study excluded pregnant women with sepsis related to early pregnancy losses, ectopic pregnancies and those in the puerperium on the basis that the physiology may not be representative of pregnancy. However, it is accepted that the physiological adaptations of pregnancy occur from conception and can persist up to 2–6 weeks post-delivery [9,10]. Furthermore, a significant proportion of maternal sepsis related morbidity and mortality occurs in the first trimester and, notably, in the puerperium [11]. As such, this study may not have accurately captured the full range of clinical outcomes seen in this population. In any event, given the heterogenous nature of sepsis there is a considerable argument for stratifying the approach to patient populations, beginning with research objectives. A “precision medicine” approach may prove pivotal in meeting these objectives. Elucidating the unique immune milieu of pregnancy and the puerperium in order to ascertain the distinct nature of the maternal response to infection may reveal potential targets for immune modulation with the aim of developing therapies.

### 2.2. Global Action

National audits, such as those produced by the Centre for Maternal and Child Enquiries (CMACE) in the United Kingdom (UK) have helped highlight the prevalence and impact of maternal sepsis as well as identifying that a number of sepsis-related maternal deaths are preventable. In 2011, CMACE reported sepsis to be the leading direct cause of death in pregnancy and the puerperium during the triennium of 2006–2008 [12]. Since then, concerted efforts have been made to tackle this head-on through public health campaigns and the development of comprehensive and accessible clinical pathways [13,14,15]. Accordingly, the last decade has seen improvements made in the diagnosis and treatment of severe infection in pregnancy and the puerperium, as demonstrated in subsequent audit cycles, which have revealed a decline in sepsis-related mortality rates. The momentum needs to be maintained; as recognised by the World Health Organisation (WHO) which, in 2017, published a resolution to recommend that member states incorporate mechanisms to improve the prevention, diagnosis and management of sepsis, including addressing infection control strategies [16].

The downward trend in UK maternal sepsis-related mortality rates needs to be replicated globally but barriers to achieving this include disparities in access to healthcare and the heterogeneous presentation of sepsis. The latter contributes to delayed diagnoses and development of septic shock. In such cases, standard management of antimicrobials and fluid resuscitation are often no longer adequate, and escalation to level 3 care for ventilatory and circulatory support is required. Hospital mortality rates at this level exceed 40% [17].

Current research is focused on unravelling the complex pathophysiology of sepsis and developing more sophisticated tools for diagnosis, as well as novel adjuvant therapies. Applying a precision medicine-based approach to this will ensure that there is receptivity to identifying any variations in the host response to infection of specific patient populations.

### 2.3. Features of the Host and Common Pathogens

Case fatality rates of sepsis have been reported to be as high as 8% in the maternal pregnant population [16,18] and morbidity is significant in survivors with an estimated morbidity/mortality ratio of 50:1 [19,20]. Genital tract sepsis is currently the fourth leading cause of direct maternal death in the UK [1]. This has seen some improvement but this is probably due to greater vigilance and a consequence of changes made to care pathways for recognition and management of the sick patient. Although evidence for increased susceptibility to infection is limited [21,22], pregnant women do experience a rapid progression to severe sepsis and septic shock, particularly in association with group A Streptococcal infection [18]. Prospective studies in the UK have suggested that labour and the puerperium may carry a 2–3-fold increase in the risk of sepsis compared to the antenatal period [11] while, in low and middle income countries, rates of fatality after puerperal infection can be as high as 50% [23]. In the puerperium there is an association between development of sepsis and operative births (Caesarean and instrumental deliveries) as well as peripartum interventions, such as intrauterine tamponade for massive obstetric haemorrhage [20,24,25].

Certain pathogens predominate. In maternal sepsis, *Escherichia coli* and group B *Streptococcus* (GBS) are the most common bacterial pathogens, but the most severe outcomes are associated with *E. coli* and group A *Streptococcus* (GAS) [19]. In fact, there appears to be a greater propensity (by 20–100 fold) for group A and group B streptococcal bacteraemia in pregnant women than non-pregnant women [26,27].

Also, of particular note, influenza pandemics appear to disproportionately affect pregnant women when compared to age-matched non-pregnant females. During the 2009 H1N1 “Swine flu” outbreak, there was a disproportionate mortality rate amongst pregnant women. Despite all pregnant women constituting 1% of the global population, 6% of the patients who died, during this outbreak, were pregnant. Similar trends were seen in earlier outbreaks [28]. The Asian flu pandemic of 1957–1958, caused by H2N2 influenza A, was associated with significant maternal mortality. Reports from the Minnesotan Department of Health detail that the virus was the principal cause of maternal mortality in the state, responsible for 19% of maternal deaths. Freeman et al. highlight that 50% of the women of reproductive age killed by the virus during the pandemic were pregnant [29].

Parasitic infections, such as malaria, can be devastating to the mother (resulting in severe sepsis and maternal death) and to the fetus (miscarriage, stillbirth, pre-term birth, fetal growth restriction) [30]. Fungal infections are a rare cause of sepsis in the immunocompetent pregnant woman, but pregnancy may increase susceptibility to some fungal infections, such as vulvo-vaginal candidiasis (which can occur in up to 20% of pregnant women). It is thought that oestrogen may increase adherence of yeast cells to vaginal epithelial cells, thus promoting colonisation which, when coupled with the increased glycogen content of the vagina, may aid proliferation of yeast cells (blastospores) and transformation to their more virulent filamentous forms (hyphae and pseudohyphae) [31,32]. Furthermore, although coccidioidomycosis infection is not increased in pregnant women who are immunocompetent, an increased severity of disease has been seen, with a greater risk of disseminated disease seen in the third trimester and early postpartum period [31]. This has been attributed to both a depressed cellular immunity to *Coccidioides* species in pregnancy as well as altered levels of 17β oestradiol and progesterone [33]. These reports support greater severity of clinical outcome in pregnant women. Further, infection has been associated with adverse outcomes for the baby, including miscarriage, stillbirth and pre-term birth [34,35,36].

### 2.4. Pathophysiology of Sepsis

Sepsis can involve any system in the body and in severe forms is associated with irreversible multi-organ failure and ultimately death [37]. The pathogenesis is highly complex and incompletely understood [38]. Establishing a universal definition of what constitutes “severe” infection is crucial for ensuring global uniformity amongst the medical community, for facilitating accurate prevalence studies (to guide health strategy) and to delineate precise criteria for study recruitment and outcome measures.

In 2016, an updated definition of sepsis and septic shock, Sepsis-3, was published [17]. Defining sepsis as “life-threatening organ dysfunction caused by a dysregulated host response to infection” [17], the consensus produced a bedside tool for prognosis and diagnosis of sepsis, validated in the general population [17]. This tool was not validated in the obstetric population [23,39] and so in 2017 the Global Maternal Sepsis Study (GLOSS) was set up by the WHO to address this [39]. This study aims to establish obstetric-appropriate sepsis identification criteria applicable for use in high, middle and low resource countries. The analysis, currently underway, aims to inform epidemiological studies and the development of strategies for prevention, early diagnosis and effective management.

In pregnancy and the puerperium, maternal physiological and immunological adaptations, designed to facilitate development of the fetus, may impair the maternal capacity to respond to infection [22]. Key physiological changes, which occur to promote the maintenance of a healthy pregnancy, mimic those of early sepsis, making diagnosis challenging [40]. For example, both respiratory rate and heart rate are increased, and blood pressure decreased, in the healthy pregnant patient when compared to the healthy female that is not pregnant. However, these are the current benchmarks used to identify the patient with suspected sepsis and septic shock [40]. This has led to the development of a modified early warning score in obstetrics that is currently used in clinical practice; and, more recently, the Society of Obstetric Medicine in Australia and New Zealand (SOMANZ) have adapted the Sepsis-3 qSOFA and SOFA scores (quick sequential (sepsis-related) organ failure assessment and sequential (sepsis-related) organ failure assessment) to help triage the obstetric patient (Table 1 and Table 2) [41]. Whether the obstetrically modified SOFA score performs more accurately in obstetric patients than the SOFA score developed for the general population remains to be determined.

The Shock index (SI), a ratio of heart rate to systolic blood pressure, has been used as a prognostic tool for severity of clinical outcome in the critically ill in the general population and a recent study demonstrated that the “worst” recorded SI, has a good negative predictive value for maternal death (96.7%) in the context of sepsis. Given that both parameters, pulse and systolic blood pressure, are altered in pregnancy, such a ratio may be a useful measure to compare haemodynamic change in the pregnant and non-pregnant patients. Further studies are required to determine its clinical value [42]. There is a growing interest in establishing the variations to the sepsis response in pregnancy and the puerperium at an immunological and cardiovascular level as this may contribute to delayed detection as well as the speed of progression and severity of disease in these patients.

Current sepsis management is based on extrapolation of non-pregnant sepsis scoring systems, investigations and treatment. We should establish an evidence-based practice for obstetric patients. The research objectives, reflecting those of sepsis research performed in the general population, should include (1) development of early diagnostic tools: based on obstetric physiological parameters and biomarkers; (2) to understand how the response to infection in pregnancy is different from non-pregnant women in order to discover pregnancy specific therapeutic approaches; (3) understand why pregnant women seem to be more susceptible to certain bacteria, GAS or GBS for example; (4) develop predictive algorithms to identify women at greater risk of sepsis who would benefit from prophylactic antibiotic treatment.

### 2.5. Precision Medicine

Multiple factors influence the complexity of the response to infection, including the organ-specific site of infection, host co-morbidities and pathogenicity of the infecting organism. In healthy pregnancy, the physiological adaptations that occur create a “new normal” and organ dysfunction cannot be defined on the basis of non-pregnant physiological variables.

The evidence on which we base our practice of medicine is evolving rapidly. Largely population based in the past, a shift to an individualised approach to clinical care (“precision medicine”) is being embraced due to the known interaction between the individual, their environment and the potential impact this has on the heterogeneity of disease phenotype [43]. We know pregnancy is a unique physiological state and therefore its interaction with disease should be investigated with this in mind. Obstetric medicine has traditionally benefited from considerable data generated from large epidemiological studies and randomised clinical trials such as in the current management of pre-eclampsia and fetal neuroprotection [44,45]. Likewise, improved knowledge of the human genome has led to advances in determining gene mutations associated with specific gynaecological malignancies. For example, Bevacizumab, a monoclonal antibody targeting VEGF (vascular endothelial growth factor) overexpression that inhibits neovascularisation and tumour growth has been shown to improve the progression-free survival (although not overall survival) when used as an adjunct to chemotherapy in ovarian cancer [46,47].

#### 2.5.1. Modifying Early Warning Scores for the Obstetric Patient

The modified early warning score (MEWS) observation chart of key physiological characteristics modified for the obstetric patient (advocated by the Confidential Enquiry into Maternal and Child Health, CEMACH, 2003–2005) [48] is a helpful screening tool for identifying the ill patient, but is notoriously non-specific. A validation study published in 2012 demonstrated an all-cause obstetric morbidity sensitivity of 89% and specificity of 79% [49]. Albright et al developed a prognostic tool to identify the obstetric patient with sepsis requiring admission to the intensive care unit (ICU) [50]. Using the traditional physiological parameters of an early warning score, they incorporated variables obtained from blood test results. The variables were scored according to variance from normal and a composite score of ≥6 would predict ICU admission with increased performance when compared with each variable individually and to two other early warning scores used in the emergency department in the US [50]. Perhaps the way forward is a composite assessment of the potentially septic patient consisting of physiological variables combined with better performing biomarkers, discussed later in this review, and should be the focus of future studies. 

#### 2.5.2. Animal Research Models of Pregnancy 

Animal models can help us understand how pregnancy alters the response to sepsis. Lipopolysaccharide (LPS), often used to simulate infection, is a component of the outer cell membrane of Gram-negative bacteria that has been implicated in the development of septic shock in humans. In a recent study, Zollner et al. [51], administered LPS and compared the response of the innate immune and cardiovascular systems in the pregnant and non-pregnant female mouse. Mortality was significantly greater in pregnancy, associated with a more profound hypotensive response. These findings were reproduced using a second model of infection, where polymicrobial sepsis was induced by caecal ligation and puncture. This model showed again that pregnancy was associated with a more marked hypotensive response and greater mortality [51]. In the group’s latest study, the administration of a broad-spectrum antibiotic and a vascular smooth muscle specific inhibitor of nitric oxide synthesis was associated with improved outcomes [52]. Hypotheses attributing these to the unique nature of the obstetric immune and cardiovascular milieu are prevalent but are yet to be validated.

#### 2.5.3. The Next Phase: Personalised Medicine for the Obstetric Patient

A number of recent studies, performed in the general population, have demonstrated a link between distinct molecular signatures and sepsis illness severity, clinical outcome and response to intervention [53]. Similar work could provide the basis for a personalised, more successful, approach to the diagnosis and management of obstetric patients. 

### 2.6. Immunology in the Obstetric Patient

Changes in the maternal immune system occur to allow the development of a healthy pregnancy. Traditionally, it has been thought that a shift from Th1 to Th2 dominance occurs to mediate fetal tolerance, while increasing the susceptibility to certain infections e.g., listeria, malaria. Interestingly, flu vaccination studies have demonstrated that Th2 predominance is associated with better antibody production in pregnancy, compared to the non-pregnant, and a suggestion that this may vary with gestation [54,55]. However, despite the earliest hypotheses of the 1950s [56], suggesting maternal immune tolerance, a lack of clarity persists as to what immune changes occur and whether they are predominantly pro-inflammatory or anti-inflammatory, activated or suppressed [57,58,59]. The non-committal term “immunomodulation” of pregnancy is often preferred, as the reality is likely to be more complex than a straightforward binary one. Indeed, there is evidence to suggest gestation may dictate a transitional inflammatory environment from “pro” to “anti” and back to “pro” again with advancing trimesters [58,60,61]. Likewise, different components of the immune system may be activated (e.g., innate) to compensate for modulation of the adaptive system (e.g., expansion of immunosuppressive T regulatory cells). In fact, the true status of the immune system will be affected by both internal and external influences that modulate the maternal immune response. These include exposure to fetal/paternal antigens, pregnancy hormones such as oestrogen and progesterone, and externally derived foreign antigens derived from pathogens and vaccines [61,62,63,64,65,66]. Furthermore, the influence that all of these immune-modulators will have on pregnancy will undoubtedly vary with gestation and this is represented in longitudinal immune-phenotype research studies. Graham et al demonstrated, in a longitudinal study of healthy pregnancy, an increased constitutive expression of anti-inflammatory cytokines when compared to a year postpartum [57]. These changes, seen from as early as the second trimester, increased with progressive gestation. A reciprocal decrease in pro-inflammatory cytokine expression was also seen during pregnancy in comparison to the postpartum status. Progesterone may mediate some of these observed effects with its anti-proliferative, anti-inflammatory and anti-cytotoxic actions [67,68,69,70,71]. In fact, this is supported by studies which show progesterone supplementation in pregnancy suppressing both cytotoxic and pro-inflammatory immune responses [61,69] and the reverse effects have been seen with progesterone antagonism (mifepristone) [61,72,73].

It is important to correlate phenotypic and functional immune findings to determine how these play a role in vivo in pregnancy-related pathology such as severe maternal infection, while remembering that this only provides a snapshot of the immune environment. As labour is a point in pregnancy when mothers are at high risk from sepsis this may be a good starting point for research [61,74]. It is well established that labour is an inflammatory process, which may be driven by fetal antigen exposure and potent innate immune stimuli [75,76]. Inflammation per se can lead to the release of mitochondrial deoxyribonucleic acid (DNA) due to tissue necrosis as well as placental derived microparticles, which can interact with Toll-like receptors (TLRs) on immune cells to drive inflammatory signals, contributing further to inflammation occurring during parturition [77,78]. Here, there is a risk that if inflammation occurs unchecked, that it may lead to increased maternal and neonatal morbidity in the event of sepsis. Interestingly, monocyte cytokine secretion, induced by placental microparticles, can be prevented with the use of TLR inhibitors [77]. Classically suppressive “regulatory” T cells (Tregs), which are abundant in pregnancy, provide a “stop signal” during an immune response and have been shown to have a protective effect in acute sepsis [79,80,81,82,83,84,85,86,87,88,89,90,91]. However, there is emerging evidence that these Tregs may be dysfunctional during human labour [82] and this dysfunction may lead to an exaggerated inflammatory response during parturition and in the puerperium. Furthermore, these findings are reflected in the fetal immune system, particularly during severe chorioamnionitis, where activated pro-inflammatory fetal T cells that migrate to the maternal circulation have been shown to potentiate maternal inflammatory processes further [83]. Therefore, exposure to pathogens, as well as the overall degree of immune modulation controlling immune responses at specific timepoints during pregnancy, will have an impact on a mother’s ability to respond to pathogenic microbes and viruses. 

Using animal models, researchers have developed experiments to reduce systemic inflammation as it is thought excessive immune cell trafficking may contribute to maternal morbidity in sepsis, rather than ineffective bacterial clearance. Using a murine model of polymicrobial sepsis in pregnancy, C-C chemokine receptor type 2 (CCR2), which is a monocyte chemoattractant receptor, knockout mice have reduced inflammatory cell trafficking, but increased bacteraemia and worse survival [52]. The same group have also shown that pregnancy in the mouse is associated with a more marked hypotensive response to LPS [51]. Therefore, cardiovascular dysfunction in sepsis may be an important contributor to poor maternal outcomes. The cellular necrosis and damage that occurs during severe sepsis leads to the release of various damage-associated molecules that bind to pattern-recognition receptors expressed on some tissues and leukocytes that activate the innate immune system [84]. Unfortunately, the normal balanced inflammatory immune response is dysregulated in sepsis due to a bias favouring widespread cytokine production, referred to as “a cytokine storm”, coupled with the generation of reactive oxygen and nitrogen species that ultimately lead to an amplified inflammatory reaction [84]. On the cardiovascular system, these molecules attenuate adrenergic responses and mediate cardiomyocyte injury. 

### 2.7. The Cardiovascular System and the Obstetric Patient

Significant morbidity is associated with the cardiovascular changes that occur during septic shock [84] and current management targeting these changes include vasopressors, inotropes and vascular compartment filling with fluids, in order to maintain optimum perfusion to vital organs. Endothelium-derived nitric oxide (NO) is up-regulated in sepsis and plays a vital role in regulation of vascular tone, smooth muscle relaxation and the vasodilatation that leads to shock [85]. When this is excessive, high levels of NO induce severe hypotension via loss of vasomotor tone. In pregnancy, abundant prostaglandins and NO (up-regulated by oestradiol) are implicated in the physiological adaptations that occur to support the developing fetus [9]. These changes may make pregnant women more susceptible to hypotension in response to infection, rapidly developing septic shock, tissue hypoperfusion and organ dysfunction. 

Troponin T, I and B-type natriuretic peptide (BNP) serum levels, are unchanged in healthy pregnancy, but have been shown to be elevated in observational studies of sepsis-associated myocardial depression in adults and children and may serve as prognostic markers of sepsis outcomes [86,87,88]. Severe sepsis also depresses myocardial function through the action of tumour necrosis factor α (TNF-α) which, combined with the fall in preload secondary to vasodilatation, leads to a marked fall in cardiac output. During pregnancy, cardiac output is increased, in part due to the reduction in afterload, but also through increased myocardial function [89]. The changes in the cardio-vascular system added to dysfunction of the microcirculation and the inhibitory effect of NO on mitochondrial function may all contribute to the development of life-threatening metabolic and lactic acidosis. However, in pregnancy, pregnancy-induced changes in cardiovascular physiology and capillary function may alter the impact of sepsis.

Echocardiography has the potential to aid in the diagnosis of sepsis-induced myocardial depression in the clinical setting, although diagnostic criteria remain to be determined [85,87]. Whilst ejection fraction is depressed in sepsis-induced cardiomyopathy, it is not very sensitive as it is influenced by fluctuations in afterload and preload [90,91]. As such, tissue Doppler imaging (TDI) which measures myocardial velocities throughout the cardiac cycle may prove to be a better tool to predict sepsis outcome, with evidence to suggest that the myocardial performance index (MPI) can provide a more accurate representation of both systolic and diastolic cardiac function in pathological states [85]. The MPI is defined as:

Isovolumetric contraction time + isovolumetric relaxation time/Ejection time

MPI was developed as a risk stratification tool and has been validated in a number of disease states, such as myocardial infarction. Its advantage is that it is load independent with shorter MPI values correlating with better global cardiac function [91].

Bamfo et al. determined reference ranges for TDI during healthy pregnancy and found that changes to the MPI are comparable with either an increased afterload or reduced contractility [92]. Furthermore, they determined that diastolic function may be the best prognostic feature of aberrant cardiac function. Further work is therefore required to determine the feasibility of applying this technique in pregnancy. 

### 2.8. Potential Targets for Bench to Bedside Diagnostics

#### 2.8.1. Tools for Improved Antimicrobial Stewardship

The most important step in managing a systemic inflammatory response is defining the cause and initiating appropriate treatment; with an infective aetiology, this means administering the correct antibiotic. Current methods have a poor specificity and sensitivity, with a causative agent being identified in 50% of cases of sepsis and they are time-consuming. 

New technologies are being developed with the potential to considerably expedite organism identification and antibiotic resistance patterns, providing clinicians with real-time information to guide antibiotic treatment; ensuring immediate appropriate treatment with the promise of reduced antibiotic resistance patterns. 

DNA sequencing is a more robust method of bacterial identification [93] than the more widely used conventional method of microscopy and culture of microbiological samples. The bacterial 16S ribosomal ribonucleic acid (rRNA) gene is a hypervariable DNA sequence of approximately 1500 base pairs that codes for 16S rRNA (a catalytic RNA, which forms part of the 30S ribosomal subunit) and is a molecular marker unique to different bacteria [93]. It has been applied to the identification of bacteria in clinical samples that are difficult to culture but is rarely available as a point of care test.

Multiplex polymerase chain reaction (PCR) uses this technology and can be used to identify multiple pathogens and their resistance genes, from both positive blood cultures and whole blood samples. The National Institute for Health and Care Excellence (NICE) advocates its use for the diagnosis of *Neisseria meningitidis* in under 16-year olds [94]. However, further evaluation of its clinical performance is required before widespread implementation can be considered [95]. The technique is currently expensive, but in the long-term this may be offset by a shorter turn-around time for results, focussed treatment and shorter hospital stays. Thus, there is scope for their widespread introduction to routine diagnostics in the future. 

Bacterial and fungal organisms can also be identified by identification of their specific protein profile or “fingerprint” using a new technique in mass spectrometry, known as Matrix Assisted Laser Desorption Ionisation Time of Flight Mass Spectrometry or MALDI-TOF MS. Advantages are that it is rapid, accurate and can identify a wide spectrum of organisms from clinical isolates of positive cultures; there is also some evidence to show the accuracy of the technique in identification of organisms directly from biological fluids, e.g., urine despite the considerably lower CFU/mL. As a result, this technology has been adopted by a number of laboratories in developed countries. However, susceptibility testing by identification of resistance phenotypes using MALDI-TOF is not yet clinically applicable and therefore the sole use of MALDI-TOF is not yet feasible [96]. An additional potential barrier to its widespread use is the expense of the MALDI-TOF instrument [97].

Key advantages of results obtained by MALDI-TOF (bacteria and fungi) and PCR based methods are that they are accurate and rapid and theoretically can be applied both to positive blood cultures and whole blood. To et al. demonstrate how MALDI-TOF can be adapted to identify group B streptococcus (GBS) from vaginal swab isolates with a reduced turnaround time and with excellent specificity and sensitivity [98]. In addition to the clinical relevance of this for women who are pregnant, as referred to previously, significant neonatal morbidity and mortality is associated with this infection. The potential diagnostic applications using other biological fluids is also of particular interest for the obstetric population as the urinary tract is a common source of infection in the pregnant and puerperal patient. Likewise, making a timely diagnosis of chorioamnionitis might be facilitated by analysis of amniotic fluid using proteomic biomarkers reflecting intra-amniotic inflammation [99]. However, presently there is insufficient data to demonstrate the benefit of using these tools for amniotic fluid. With PCR, the ability to amplify microbial products also means that non-pathogenic organisms will be identified, which may complicate the diagnostic picture. 

The ultimate objective is to provide clinicians with the ability to target antibiotic treatment for the pathogen and its sensitivity, to increase efficacy of treatment and to obviate multi-antibiotic resistance. 

#### 2.8.2. “Omics Studies”—Biomarkers for the Obstetric Patient

Omics based technologies enable quantitative and qualitative analysis of the molecules involved in biological pathways. Multiple molecular levels including DNA (genome), mRNA (transcriptome), proteins (proteome), and metabolites (metabolome) can be targeted to elicit the pathophysiology of a given disease process. Having identified specific biological molecules using these techniques, their influence in sepsis research can be assessed in the context of their role in the immune and metabolic responses to disease and enable scientists to pinpoint aberrant steps in biological pathways that may contribute to disease. 

As sepsis is such a heterogenous condition with multi-systemic impact, it is unlikely that a single biomarker would have significant sensitivity and specificity for the early diagnosis of sepsis; using multiple biomarkers may increase the yield and omic technologies may be a way to achieve this [100]. The “omics” approach has the potential to help identify “signature” biological markers that can be developed into clinical tools for diagnosis and prognosis. Additionally, the field holds promise in guiding clinicians in the escalation and de-escalation of appropriate therapy (theragnosis) whereby monitoring patient response to treatment allows us to tailor therapy to the individual and enable development of novel therapies [101,102]. There is the potential for omics studies to revolutionise sepsis care [103].

Transcriptomics, in particular, has been a focus of such studies, analysing the host signalling response in sepsis. The transcriptome of immune cells from patients with sepsis theoretically could provide an early diagnostic tool for sepsis. The set of mRNA markers it represents known as the “transcriptomic signature”, may demonstrate the earliest change triggered by the infective process before translation of the relevant proteins [100]. Indeed, there is emerging evidence that a transcriptomic signature is a reflection of the host’s sepsis response, and has prognostic value, particularly regarding mortality risk, and this is independent of the causative pathogen or site of infection [104,105].

A recent study identified two distinct transcriptomic profiles of peripheral blood leucocytes amongst a subgroup of patients admitted to the Intensive Care Unit with sepsis secondary to community acquired pneumonia (CAP) [105]. These profiles correlated with different clinical phenotypes, one of which correlated with markers consistent with an immunosuppressed state (characterised by endotoxin tolerance, T cell exhaustion and downregulation of HLA class II) and was associated with higher patient mortality [105]. Thus, it can be seen that, with further development, this technology could be used for prognosis of illness severity in patients admitted to ICU with CAP. 

Another prospect of transcriptomics is the potential to distinguish bacterial from viral infections and the associated advantage of targeted antibiotic therapy. In a multi-centre prospective observational study of 370 febrile children, a disease risk score (DRS), determined by evaluation of the differential expression of a 2-transcript RNA signature, was able to distinguish bacterial from viral infection with excellent sensitivity and specificity (100% and 96% respectively). There was also good correlation of these findings when the transcript was applied to previously published datasets of RNA expression in adults and children. The transcripts of note, IF144L (interferon-induced protein 44-like) and FAM89A (family with sequence similarity 89, member A), demonstrated increased expression with viral and bacterial infections respectively [106]. The ability to clinically discriminate widespread inflammation of infectious aetiology from that which is non-infectious is another important tool for stratifying patients for the prudent use of antibiotics and studies have demonstrated that transcriptomics may be a sensitive method to do this [107,108]. The “SeptiCyte Lab“ uses a microarray panel of 4 RNA transcripts (CEACAM 4, LAMP1, PLA2G7 and PLAC8) and has demonstrated superior performance (AUROC) distinguishing systemic inflammatory response syndrome (SIRS) from sepsis, when compared to the performance of procalcitonin using whole blood samples, and is one of a number of tests that have been approved by the Food and Drug Administration (FDA) in the United States of America to aid clinical diagnosis [108,109]. Along similar lines, an RNA microarray analysis study, with quantitative reverse transcriptase-polymerase chain reaction (qRT-PCR) validation, demonstrated that the ratio of gene expression of FAIM3:PLAC8 can be used to determine whether respiratory distress in ITU admissions is secondary to community acquired pneumonia or a non-infectious cause. No prognostic ability was demonstrated [110]. 

Analysis of the genomic expression of peripheral blood white cells in sepsis in pregnancy may demonstrate activation or suppression of specific pathways, which could guide an approach to “precision medicine”. Differential gene expression may be associated with known features of immune tolerance, nitric oxide synthesis, cytokine production or reveal new pathways unique to this population. All of which are potential targets for novel therapies. 

In parallel, a growing number of studies, including that of Ferrario et al [101,111], demonstrate that plasma metabolite levels reflect the severity of sepsis and septic shock and have been associated with the risk of mortality [112]. Further, the metabolite signature or metabolome may even prove useful in guiding response to therapy in the acute phase of sepsis [113]. Using this technique, it may be possible to determine metabolic profiles of plasma and urine in pregnant women with suspected infection that are associated with the development of severe sepsis to further our understanding of the pathophysiology of the syndrome. Urine samples, in particular, also have the potential to provide non-invasive point of care testing to facilitate early diagnosis and treatment [114]. 

A hugely exciting prospective study is the Bioresource in Adult Infectious Diseases (BioAID), a multicentre study in the UK, collecting biological samples from patients who present to the Emergency Department with suspected infection [115]. Key objectives of the study include analysis of the immunological, proteomic, metabolomic and transcriptomic gene signatures of these patients with the aim of increasing our understanding of the characteristics of host susceptibility to infection and important features of the host-pathogen interaction which may influence the trajectory of disease severity. The resulting data has huge potential to guide biomarker development for the early diagnosis of bacterial infection. Such a study provides a unique opportunity to include pregnant and parturient women presenting to obstetric departments with suspected infection. 

Thus, in addition to our current range of assessment tools and investigations, perhaps we can hope to improve our diagnosis and management of sepsis with the addition of immunological point of care tests, echocardiography and omics technologies. 

### 2.9. Ethical Considerations

High quality studies support the practice of evidence-based medicine for obstetric patients. Barriers to this have predominantly been researcher-led as pregnancy has traditionally been an exclusion criterion for the majority of studies. While this perception may persist in some interventional drug trials due to concerns about potential teratogenicity or toxicity, more studies are recruiting pregnant participants. This is key as a recent systematic review [116] showed that as many as 84%–99% of pregnant women may be using medicines which lack substantial safety, efficacy or fetal risk data and that this is a direct result of the under-representation of pregnant women in clinical research. Additionally, patient-led barriers to participation in research include accessibility and convenience. For example, pregnant patients may already be mothers and childcare for existing children may be a barrier to attending frequent research visits [117]. 

While an exciting prospect, omics research will inevitably generate large quantities of bioinformatic data, which raises a number of ethical issues. For example, genomic data, unique to the individual, is potentially identifiable despite anonymisation at recruitment and needs robust regulation to protect participants. Furthermore, due to the complex data obtained, requiring a range of expertise, data sharing across international networks is desirable. Such data needs to be appropriately stored to allow meaningful conclusions to be made without compromising patient identity. Another inevitable concern is that of incidental findings of aberrant genes, which may or may not be associated with disease. While it may be in the patient’s best interest to be informed, a method of identifying the participant would need to be in place. Clear consent is important and clinically sensitive data would need to be managed carefully. Regulation of this data must ensure that the participant’s interests remain at the forefront.

## 3. Conclusions

Sepsis, defined as the dysregulated host response to infection, can involve almost every system in the body and in severe forms is associated with irreversible multi-organ failure and, ultimately, death. The pathogenesis is highly complex and incompletely understood. 

In pregnancy and the puerperium, the maternal immunological and cardiovascular adaptations, designed to facilitate the development of the fetus, may impair the maternal capacity to respond to infection. The heterogenous nature of sepsis reinforces the need for a precision-based approach. Current sepsis research encompasses genetics, molecular biology, immunology and the cardiovascular system in search of improved diagnostic tests and therapeutic interventions. A proactive approach to the inclusion of pregnant and puerperal patients in basic science and clinical research studies is critical.

Heightened awareness of the clinical presentation of sepsis and the potential severity of clinical outcomes, amongst both the public, via successful global and national educational campaigns, and amongst health care professionals, through concerted health strategies and guidelines, will help to reduce the maternal morbidity and mortality but, in parallel, research and development is essential for the introduction of precision medicine in obstetrics.

## Figures and Tables

**Table 1:** Obstetrically modified qSOFA score.

Parameter	Score	
	0	1
Systolic Blood Pressure(mmHg)	≥90	<90
Respiratory Rate	<25 breaths/minute	≥25 breaths/minute
Altered Mentation	Alert	Not alert

qSOFA = quick Sequential (sepsis-related) Organ Failure Assessment; mmHg = millimetres of mercury). Adapted with permission from the SOMANZ guidelines for the investigation and management of sepsis in pregnancy, this table allows a rapid clinical assessment before investigations are available, to quickly identify the critically ill obstetric patient. Altered mentation alone or a score of 2 or more may be associated with significant morbidity in a pregnant patient with suspected sepsis and should trigger senior medical input (obstetrican/ physician) [35].

**Table 2:** Obstetrically modified SOFA score.

System Parameter	Score		
	0	1	2
**Respiration**			
PaO_2_/FiO_2_ (mmHg)	≥400	300 to <400	<300
**Coagulation**			
Platelets (×10^6^/L)	≥150	100–150	<100
**Liver**			
Bilirubin (µmol/L)	≤20	20–32	>32
**Cardiovascular**			
Mean arterial pressure (mmHg)	MAP ≥70	MAP <70	Vasopressors required
**Central Nervous System**	Alert	Rousable by voice	Rousable by pain
**Renal**			
Creatinine (µmol/L)	≤90	90–120	>120

PaO_2_ = partial pressure of oxygen; FIO_2_ = fraction of inspired oxygen (in mmHg, millimetres of mercury, expressed as a decimal); MAP = mean arterial pressure (in mmHg); SOFA = Sequential (sepsis-related) Organ Failure Assessment. Adapted with permission from the SOMANZ guidelines for the investigation and management of sepsis in pregnancy, this table demonstrates a sepsis-related scoring system which uses pregnancy-specific physiological variables to identify the critically ill obstetric patient. In the general population, an acute change in the SOFA score from baseline of ≥2 (where the baseline in the healthy general population can be assumed to be 0 if unknown) has been associated with an increased risk of in-hospital mortality. Further studies are required to ascertain the validity of this data in the obstetric population [35].
